# Self-Efficacy, Self-Management and Use of Smartphone Apps for Low-Back Pain

**DOI:** 10.1155/prm/5584106

**Published:** 2025-12-04

**Authors:** Claudia Didyk, Belinda Lange, Lucy Kate Lewis

**Affiliations:** Caring Futures Institute, College of Nursing and Health Sciences, Flinders University, Adelaide, South Australia, Australia

**Keywords:** behaviour change, consumers, low back pain, personality traits, self-efficacy, self-management, smartphone apps

## Abstract

**Aim:**

To explore the self-efficacy and self-management practises of people with low back pain (LBP), including associations between participant characteristics, self-efficacy and self-management. The secondary aim was to describe the characteristics of people with LBP who use smartphone apps for self-management, including app preferences.

**Methods:**

Prospective cross-sectional online survey of Australian adults with current or previous LBP. Descriptive statistics were completed for all variables. Associations between participant characteristics, self-efficacy and self-management were explored through linear regression. Alpha was 0.05.

**Results:**

A total of 136 survey responses were included (55.5 ± 14.5 years, 74% female). Most participants (93%) had LBP at the time of the survey and reported constant or daily (58%) pain of moderate severity. Nearly all participants managed their LBP on their own (91%), with the most frequently used self-management options including exercise (47%), advice from health professionals (38%) and pharmacological management (37%). Most self-managed either moderately (35%) or fairly (32%) well, with a mean self-management score of 11.9 ± 4.0 out of 20 and a mean self-efficacy score of 3.6 (±0.8), well above mid-range of 2.5. Lower socioeconomic status and higher scores in personality traits were associated with higher self-efficacy (*p* < 0.05). Longer duration and increased recurrence of LBP were associated with lower self-efficacy (*p* < 0.05). Participants with higher self-management scores were female, older, with higher scores in personality traits aside from agreeableness and lower severity of LBP (*p* < 0.05). Seventeen participants (13%) used apps.

**Conclusion:**

Most adults with LBP self-managed well and had above-average self-efficacy. Smartphone app use was limited, with lack of knowledge a perceived barrier (59%, *n* = 73).

## 1. Introduction

Low back pain (LBP) is a global health and economic concern and is rated as the number one cause of disability worldwide [1]. It occurs in all age groups, but most commonly in working age [1], in all countries, regardless of income level, and often without a specific nociceptive cause [2]. Although recovery can be rapid, recurrence is common [3]. In Australia, an estimated four million (16%) people experienced back problems (related to the cervical, thoracic or lumbar spine) in 2022, with LBP the most common musculoskeletal condition [4, 5]. The burden of LBP is increasing with an ageing population, high inflation, rising healthcare usage post pandemic and increasing global private health costs [6]. Innovative low-cost, scalable self-management options are required to manage the burden of LBP.

Self-management requires continuous self-regulation [7] and is a key recommendation in current LBP guidelines, together with active rehabilitation and exercise [8, 9]. Self-management involves consumer participation in decision making [8, 10–12], and monitoring and management of treatment, physical symptoms and psychosocial requirements of the condition [7]. Self-management has been shown to reduce pain intensity and disability in people with LBP [13]. Targeted self-management interventions, personalised to specific groups of people may be the most beneficial, with small to medium effects reported [14]. Modifiable biopsychosocial factors, such as physical activity, disability, catastrophising, kinesiophobia and depression, impact self-management [14]. Additionally, self-management, health behaviour [15, 16] and a person's openness to change [17–19] may be impacted by ethnic differences [3] and personality traits. Disability resulting from LBP varies and is influenced by social norms and local health care [3]. Therefore, individual preferences, needs, personality traits, self-efficacy and self-management capacity, require consideration in behaviour change interventions [19].

Smartphones applications (apps) have potential to facilitate self-management of LBP [20], providing a cost-effective option to improve access to health management guidance and monitoring [21–25]. The Covid-19 pandemic necessitated increased use of digital technology in health [26–28], as consumers experienced difficulties accessing health care [29]. Smartphones are ubiquitous in developed countries [30] and have capacity for great reach at low cost as scalable self-management interventions [31]. Apps have capacity to improve pain [32–34] and disability outcomes [35] or at least provide similar pain outcomes to physiotherapy for people with LBP [29, 36]. Despite consumers being willing to use digital health technologies [27] to more actively manage their health [26], apps continue to be underutilised [37].

International studies have explored self-management of LBP to address current clinical practise and increase self-management [14, 38–40]. In Australia, studies have explored the needs and experiences of people with LBP in primary care [41] and beyond [42]. Self-efficacy and self-management of people with LBP have not been explored in the Australian context, and we currently do not know whether and how people with LBP in Australia are using apps for self-management. There is a clear need to better understand the self-efficacy and self-management practises of people with LBP and their use of apps for self-management. This information may guide the development of targeted and scalable interventions to improve self-management of LBP.

The primary aim of this study was to explore the self-efficacy and self-management practises of people with LBP, including associations between participant characteristics, self-efficacy and self-management. The secondary aim was to describe the characteristics of people with LBP who use smartphone apps for self-management, including app preferences.

## 2. Methods

### 2.1. Design

This study used a prospective cross-sectional design with an online survey (Qualtrics). Ethical approval was gained. Informed consent was gained online prior to undertaking the survey.

### 2.2. Participants

People were eligible if they resided in Australia, were aged at least 18 years, and had experienced LBP of any duration. The survey was disseminated from April to July 2022 through social media (convenience/social sampling) and relevant support groups (purposive sampling) (e.g. Back Pain, Disc Bulge/Herniation & Sciatica Support, Back Pain & Sciatica Sufferer Support).

### 2.3. Survey Instrument

A survey was developed based on existing valid and reliable instruments and pilot tested for clarity. The final survey consisted of 40 items (36 closed, 4 open).

• *Personal information* was collected, including age, gender, nationality at birth, language, highest level of education and employment status. Postcodes were used to identify socioeconomic status (SES) by using the Australian Bureau of Statistics Socioeconomic Indexes for Areas which ranks areas according to relative socioeconomic advantage and disadvantage. Three SEIFA scores were used, the disadvantage score (standardised mean of 1000, a higher score indicates higher SES), deciles (1 being the lowest and 10 the highest) and percentiles ranging from 1 (lowest) to 100 (highest) [43].
• *Personality traits* were assessed with the Ten-Item Personality Inventory (TIPI), a reliable assessment (average Cronbach *α* = 0.53) [44] of personality dimensions including: (1) extraversion, (2) agreeableness, (3) conscientiousness, (4) emotional stability and (5) openness to experience [45]. The TIPI consists of 10 personality trait pairs requiring a rating as to what extent the pair of traits apply [45]. The higher the score for each trait, the more likely a person's behaviour and thoughts reflect the characteristics of that trait [45]. The published norm scores (average adult scores based on 1813 participants) for each personality trait were applied for analyses [45].
• *Self-efficacy* was assessed using the New General Self-efficacy scale (NGSE) [46], a reliable measure (Cronbach *α* = 0.95) [47] which asks respondents to rate their level of agreement with eight self-efficacy-related statements [46]. A higher score indicates greater self-efficacy [46].
• *Low back pain information* included duration, frequency, average pain rating (11-point numeric pain rating scale, NPRS [48]) and management approaches.
• *Self-management* competence was assessed using the reliable (Cronbach *α* = 0.80) 5-item Self-Management Self-Test (SMST) [49] consisting of five questions and scored using a five-point scale [49]. A final self-management score is calculated, with a higher score indicating better self-management [49].
• *Smartphone app use and preferences* were explored. For app users, the quality of the apps they used was explored with items based on the Mobile App Rating Scale (MARS) (engagement, functionality, aesthetics and information) [50] (Omega 0.79–0.93) [51]. App potential for developing self-management survey items were based on the Self-management Support Checklist (SMS-14) [52], in the following categories: (1) self-efficacy building, (2) self-tailoring, (3) self-monitoring of symptoms, (4) goal setting and planning, (5) problem solving and (6) partnership between views of patient and clinicians. The behaviour change potential of the identified apps was assessed using the App Behaviour Change Scale (ABACUS) (knowledge and information, goals and planning, feedback and monitoring and actions) (Cronbach *α* = 0.93) [53] and the COM-B model for behaviour change (capability (C), opportunity (O) and motivation (M)—the three factors capable of changing behaviour (B)) [54]. Finally, participants were asked about the features of apps that they liked or disliked, and whether specific features helped with LBP self-management.

### 2.4. Data Management and Analysis

Survey data were exported into Microsoft Excel. Survey responses that were not located in Australia, ‘bot' responses (identified by the research team through patterns and inconsistencies in responses or provided only demographic details) were excluded. Where participants answered only the first part of a multicomponent question, the incomplete question items were treated as missing data. The final dataset was exported into the Statistical Package for Social Science (IBM SPSS) version 28.0.1.1. A sample size greater than 30 permitted the assumption of normal distribution, of continuous and approximate variables, based on the central limit theorem [55, 56] and guidance from a statistician. Descriptive analyses were completed on all variables, including means, standard deviations (SD) and ranges. Where participants answered only the first part of a multicomponent question, the incomplete question items were treated as missing data. Missing data was tested to check missingness completely at random (MCAR) and Little's MCAR test (chi-square = 145.416, *p*=0.184) suggest a randomness of missing values, unrelated to both observed and unobserved data. Therefore, missing data were omitted, and complete case analysis was used to achieve unbiased estimators. To explore associations between participant characteristics, self-efficacy and self-management, linear regression analyses were used. Smaller samples were grouped for analysis. Significance was set at 0.05, with 95% confidence intervals reported. Due to inadequate power to run app user analyses (13% of total sample, *n* = 17), descriptive statistics were used to report characteristics and behaviours of app users.

## 3. Results

There were 208 survey responses, with 73 subsequently excluded, leaving 136 responses for analysis.

### 3.1. Participant Characteristics

#### 3.1.1. Whole Sample

Participants (*n* = 136) were aged 55.5 (±14.5) years (range 18–80 years) and 74% were female (Table 1). Most participants had English as a first language (95%) and were born in Australia and New Zealand (72%) (grouped as Oceania). Four percent (*n* = 5) identified as Aboriginal or Torres Strait Islander. The mean SEIFA disadvantage score was 995.6 (±46.7) (range 906–1115) (below the standardised mean of 1000) and the mean SEIFA decile score was 6.6 (±2.4). Most participants had university-level education (56%) and 55% were not employed. Most had below norm extroversion (70%, *n* = 95), openness (64%, *n* = 87) and emotional stability (58%, *n* = 79) and above norm conscientiousness scores (51%, *n* = 69).

Most participants had access to an iPhone (65%, *n* = 81) or android phone (44%, *n* = 54) and 51% (*n* = 63) had access to an iPad or other tablet device (24%, *n* = 29). Three participants (2.2%) did not have access to any smartphone or tablet device. Most participants (87%, *n* = 111) rated themselves at least moderately confident using smartphone or tablet devices.

Most participants (93%, *n* = 127) had LBP at the time of the survey and reported constant or daily (58%, *n* = 69) pain with mean pain severity of 6.3 out of 10. Most participants reported pain lasting longer than 12 weeks (67%, *n* = 91) and experienced a recurrence of LBP constantly to daily (58%, *n* = 69) (Table 1).

### 3.2. LBP Self-Management Options

#### 3.2.1. Use

The majority of participants reported managing their LBP on their own (*n* = 124) and used exercise (*n* = 123) (Table 2). The most popular self-management options that were reported to be used frequently were exercise (47%, *n* = 59), advice from health professionals (38%, *n* = 48) and pharmacological management (37%, *n* = 47) (Table 2). Other self-management options used frequently (25%) included self-treatment such as self-adjustments, medication and passive treatments (e.g. Traditional Chinese Medicine, psychological/mindfulness therapies). The self-management options used rarely or not at all were return to work (RTW) programs (91%, *n* = 114), psychological therapies with physical programs (69%, *n* = 93) and Internet information (50%, *n* = 62).

#### 3.2.2. Effectiveness

**Figure 1:**
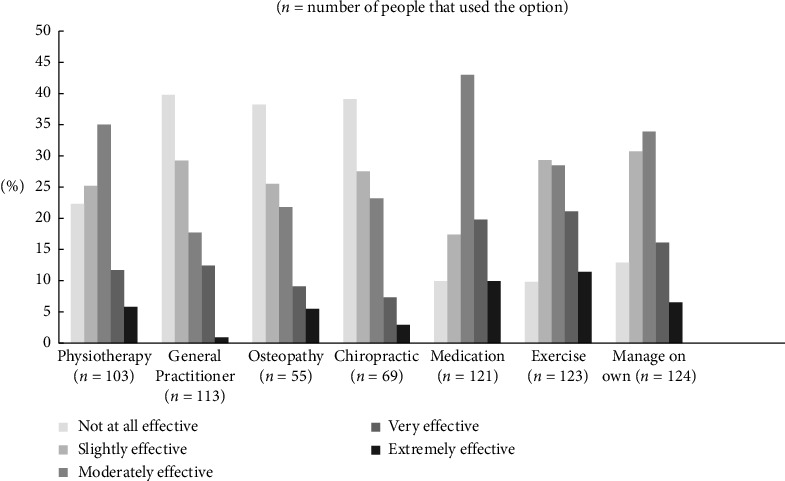
The use of different low back pain (LBP) self-management treatment options and consumer self-reported perceived effectiveness (*n* = 55–124).

Extremely effective (19%, *n* = 11) or very effective (29.3%, *n* = 17) treatments included passive treatments (e.g. massage, acupuncture/Traditional Chinese Medicine), psychological or mindfulness therapies (e.g. meditation) and a range of low-impact exercise (e.g. Pilates, yoga, swimming). In terms of perceived effectiveness (Figure 1), most participants considered medication (73%, *n* = 88), exercise (61%, *n* = 75), managing on their own (57%, *n* = 70) and physiotherapy (53%, *n* = 54) to be at least moderately effective. Treatment options that were considered not at all effective were no treatment (57%, *n* = 40) or consulting a General Practitioner (40%, *n* = 45), Chiropractor (39%, *n* = 27) or Osteopath (38%, *n* = 21).

### 3.3. Self-Efficacy and Self-Management Scores

Most participants self-managed either moderately (35%, *n* = 44) or fairly (32%, *n* = 40) well, with a mean self-management score of 11.9 (±4.0, range 3–20) out of a maximum possible score of 20, and a mean self-efficacy score above mid-range of 2.5 (3.6 ± 0.8, range 1–5) (90%, *n* = 122).

### 3.4. Associations Between Participant Characteristics and Self-Efficacy

Lower SES (SEIFA Percentile) was correlated with higher self-efficacy (*r* = −0.221) (Table 3). All personality trait scores (conscientiousness, agreeableness, openness, emotional stability and extroversion) showed a statistically significant positive correlation with self-efficacy, with higher personality trait scores associated with higher self-efficacy. Both LBP duration (*r* = −0.213) and recurrence (*r* = −0.266) were inversely associated with self-efficacy, with longer durations and increased recurrence of LBP associated with lower self-efficacy.

### 3.5. Associations Between Participant Characteristics and Self-Management

Females had higher self-management scores than males (*R*^2^ = 0.064, *F*(1, 124) = 8.486, *p* = 0.004) and increasing age was associated with higher self-management scores (*R*^2^ = 0.074, *F*(1, 124) = 9.846, *p* = 0.002) (Table 3). Higher levels of extroversion, conscientiousness, emotional stability and openness were positively associated with higher self-management scores (Table 3). There was an inverse association between LBP severity and self-management (*R*^2^ = 0.046, *F*(1, 125) = 6.077, *p* = 0.029) (Table 3). On a separate question to the SMST, people with higher self-management scores self-rated their ability to self-manage their LBP higher (*r* = 0.529, *n* = 127, *p* < 0.001).

### 3.6. Use of Smartphone Apps by People With LBP

#### 3.6.1. App User Characteristics

Seventeen of the 136 participants (13%, *n* = 17) reported using apps to manage their LBP (Table 1). The small number of participants prevented app user analyses due to inadequate power, as such descriptive statistics have been used. The main reasons for not using apps were lack of knowledge (76%, *n* = 73) and deliberate choice (24%, *n* = 23). All app users had access to a smartphone and most (83%, *n* = 14) to a tablet device. Most participants (65%, *n* = 11) were very confident using smartphone/tablets. App users had a higher level of unemployment (65%, *n* = 11), lower socioeconomic disadvantage, with a SEIFA score of 1015.4 (±41.9) (above the standardised mean of 1000) and mean SEIFA decile score of 7.6 (±2.0) when compared to the whole sample. Most app users had below norm scores for extroversion (82%, *n* = 14), agreeableness (77%, *n* = 13), openness (65%, *n* = 11) and conscientiousness (59%, *n* = 10) and above norm emotional stability (53%, *n* = 9).

All app users had LBP at the time of survey completion, with 71% (*n* = 12) reporting constant or daily LBP and 82% (*n* = 14) with pain lasting longer than 12 weeks (Table 1). Most app users reported self-managing their LBP moderately (35%, *n* = 6) or fairly (35%, *n* = 6) well, with a mean self-management score of 13.06 (±3.93, range 6–20) out of a maximum score of 20 and a mean self-efficacy score above mid-range of 2.5 (94%, *n* = 16) (3.7 ± 0.8, range 2–5).

#### 3.6.2. Self-Management and App Use

**Figure 2:**
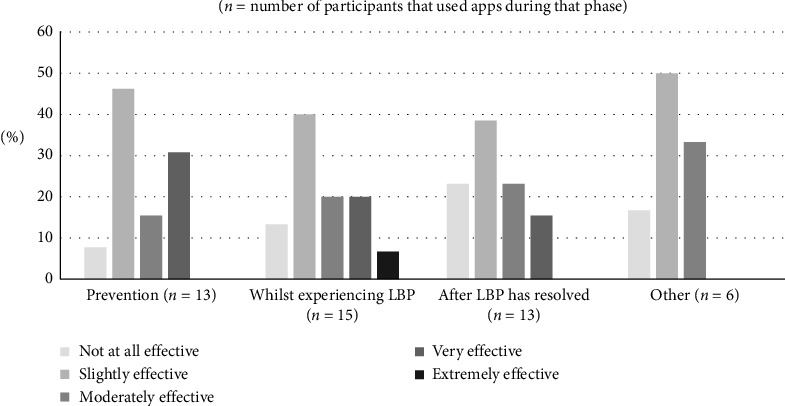
The phase of low back pain (LBP) self-management app use consumers self-reported to be most effective (*n* = 6–15).

Apps were used whilst experiencing LBP (94%, *n* = 16), for prevention (81%, *n* = 13) and after LBP had resolved (81%, *n* = 13) (Figure 2). Seventy-five percent (*n* = 6) of app users who responded reported that apps were effective in improving LBP, and at least slightly effective during prevention (92%, *n* = 13), whilst experiencing LBP (80%, *n* = 15) and after it had resolved (77%, *n* = 13). Three participants (30%) reported that they self-managed at least moderately well with LBP apps. Seven participants (70% of app users) reported that there was no change in how often they sought treatment for LBP when using apps.

### 3.7. Apps Used for LBP Self-Management

Apps used by participants included FlareDown (Logan Merriam), Microsoft OneNote (Microsoft), YouTube (Google), Back pain exercises at home (Vladimir Apps), Lower back pain exercises (Steveloper), Back pain relief yoga at home (Dr Zio), Notes (QR Scanner & QR Code Generator & Radio & Notes) and Insight Timer (Insight Network Inc). The app features most often used were meditation and relaxation (Insight Timer); tracking of symptoms, treatments and management (FlareDown); exercises (Back pain exercises at home, Lower back pain exercises, Back pain relief yoga at home) and note taking and recording (Notes).

App users reported using their chosen app for months (33%, *n* = 2) to years (67%, *n* = 4). Five participants (63%) used their chosen app daily. The behaviour change features that helped to improve LBP were motivation (67%, *n* = 4), healthy behaviour skills (67%, *n* = 4), self-management prompts (50%, *n* = 3) and example self-management behaviours (50%, *n* = 3). Most participants (83%, *n* = 5) reported that the behaviour change features that did not improve LBP were education, in-app rewards, fear of consequences, restriction of unhealthy behaviours and behavioural support options (67%, *n* = 4).

Participants agreed or strongly agreed that their chosen app provided prompts to encourage self-management (60%, *n* = 3), examples of self-management behaviours (50%, *n* = 2) and motivational language (50%, *n* = 2). Participants agreed that their chosen app provided information to promote self-management (80%, *n* = 4), behavioural support options to reduce barriers to self-management (80%, *n* = 4) and taught skills required to self-manage (50%, *n* = 2). Over 57% (*n* = 4) of the apps did not provide a reward system, consequences for not following self-management advice or behavioural restrictions.

While not all identified apps had all features that were rated, the monitoring feature of apps was reported to be important (80%, *n* = 4). Advice, goal setting, prompts and alarms, and social support features were also rated as important (50%, *n* = 2). Planning for flare-ups was the most used feature (100%, *n* = 5), followed by education and information (75%, *n* = 3), advice (67%, *n* = 2), monitoring of progress and symptoms (60%, *n* = 3) and goal setting and prompts and alarms (both 50%, *n* = 2). The least used features were personalisation (75%, *n* = 3) and social support (67%, *n* = 2).

App features that were liked by all participants using apps with that feature included: interactivity (*n* = 3), graphics (*n* = 5) and quality of information (*n* = 4). Navigation (86%, *n* = 6), gestural design (86%, *n* = 6), layout (83%, *n* = 5), visual appeal (83%, *n* = 6), accuracy of app description (83%, *n* = 5) and interest (80%, *n* = 4) were liked by over 80% of participants using apps with those features. The least liked features were entertainment (75%, *n* = 3) and visual information (67%, *n* = 4). The features that most commonly were not included in the identified apps were interactivity (57%, *n* = 4), and customisation, goals, quality of information and evidence base (43%, *n* = 3).

## 4. Discussion

This study aimed to investigate the self-efficacy and self-management practises of people with LBP, as well as explore the use and preferences for smartphone apps in self-management. Most adults in Australia reported daily, moderately severe LBP of at least 3 months duration. People with LBP had moderate self-efficacy, with higher scores associated with SES, higher personality trait scores and shorter LBP duration and recurrence. Most people with LBP self-managed moderately well, most commonly on their own, with effective self-management including medication, exercise and physiotherapy. Higher self-management scores were associated with being female, lower pain severity and higher scores on extroversion, conscientiousness, emotional stability and openness. Only a small proportion of people reported using apps to self-manage their LBP, with popular behaviour change features including monitoring, advice, goal setting, prompts and social support options.

Most people with LBP reported frequently self-managing their LBP with exercise, with low-impact exercise perceived to be at least very effective. This aligns with current guideline recommendations that encourage patient empowerment and autonomy to self-manage. Although people with LBP also perceived passive treatments, psychological and mindfulness therapies, medication and physiotherapy to be at least moderately effective, it also suggests that people with LBP may prefer to self-manage with exercise over other health care interventions. However, the choice to self-manage highlights the need to ensure adequate knowledge and support to effectively do so.

Self-efficacy is important for people with LBP, impacting recovery and predicting progression to chronicity [57–61]. Consistent with previous literature, conscientiousness, extroversion, openness, emotional stability and agreeableness were associated with higher self-efficacy [62–65]. People with stronger personality traits may have improved coping mechanisms [66] to better manage stress [67], resulting in higher self-efficacy and improved self-management. Conflicting findings in relation to SES and self-efficacy in people with LBP have been previously reported [68]. This study found a significant inverse association between SES and self-efficacy, which may partially be explained by self-efficacy mediating possible impacts of low SES [69, 70], suggesting that people with lower SES may develop resilience and coping strategies, through necessity, due to fewer supports. Practical and experience-based knowledge for managing LBP may also result in increased perceived confidence to self-manage even if with lower health literacy. Additionally, SES measurement tools may not effectively capture a person's functional access to resources, social capital and lack of broader health-related self-efficacy even if they feel confident self-managing LBP. However, the results may be skewed if the sample includes many motivated, help-seeking lower SES participants who may already possess higher self-efficacy, resulting in a selection bias that is not representative of the broader population. Effective interventions should focus on equitable access to education, employment and healthcare rather than solely aiming to boost self-efficacy which may already be high. Conversely, for those from higher SES areas interventions should focus on boosting self-efficacy by supporting intrinsic motivation and autonomy and reducing reliance on external systems. It should be noted that the average SES level in this study was very close to the standardised average in Australia [71], with further exploration required of low and high SES bands and self-efficacy. Early intervention is particularly important for people with LBP due to relationships between LBP duration, recurrence and lower self-efficacy [72].

Self-management is a key recommendation in LBP guidelines [73] to enhance a person's ability to manage day-to-day and improve quality of life [12]. People with stronger traits in conscientiousness, openness, extroversion and emotional stability self-manage better and are more likely to undertake health-promoting activities [74–76]. Those who are open to new experiences (openness), thoughtful with good impulse control (conscientiousness), sociable and assertive (extroversion) and deal better with stress and are emotionally resilient (emotional stability) [77], may be more likely to effectively self-manage. Although not found in this sample, high levels of agreeableness have been shown to improve self-management [78, 79]. In this study, people with LBP self-managed moderately well; however, in contrast to previous literature [14, 80], lower age and higher LBP severity were associated with lower self-management. This may be due to the differing methods used for data collection, however, in agreement with Banjeree et al. [14]; pain duration did not predict self-management and its change.

Despite most study participants reporting self-managing their LBP effectively on their own, the number using apps to aid self-management approaches was lower than anticipated. Interestingly, those who used apps to self-manage their LBP had below norm personality traits aside from emotional stability, which contrasts with the finding that higher personality traits are associated with improved self-management and self-efficacy in the whole sample. Although personality traits are rarely cited as reasons for choosing an app, they may play a significant role in understanding the relationship between these traits and app usage. Higher levels of some personality traits in the general population are associated with increased use of physical activity apps such as Strava or Fitbit [81]. Many general physical activity apps emphasise the importance of social support features, such as sharing on social media platforms, to encourage adherence to app use and increase physical activity [81]. Interestingly, we found that most of the app users did not place importance on social support features, and few used them if they were available, suggesting different goals and needs to the general population.

LBP interventions should encourage behaviours that improve health outcomes by encouraging self-management [82]. In this study, self-management and behaviour change features were often not provided in the identified apps which aligns with the current literature [83]. App users had higher mean self-management scores than the whole sample but slightly lower median self-efficacy scores. It is possible that those with higher personality trait scores, self-efficacy and self-management who did not use apps self-managed well without the need for adjunct self-management options such as apps. In contrast, those with lower personality trait scores and associated lower self-efficacy may require apps as an adjunct self-management option to assist with improving self-management.

Apps appear to be used by a small proportion of people with LBP as an adjunct to other management options. App users placed importance on the monitoring of progress and planning for flare-ups in apps, and liked app quality functionality and aesthetic features. App developers should include these features to encourage self-management. There is a need for health professionals such as physiotherapists, to understand the importance of self-efficacy and self-management for people with LBP. We currently do not know how health professionals use apps for clients with LBP, or their understanding of the role of apps in self-management. Health professionals could include personality trait assessments when considering recommending apps. Additionally, app use could be embedded in treatment plans, with health professionals ensuring comprehension and appropriate use of app features to enhance adherence. Future research may explore potential correlation between self-management behaviours and consumer choice between active and passive health professional care. Additionally, future qualitative research is needed to understand how personality traits might influence engagement with self-management apps in people with LBP. Assessment of app quality and self-management and behaviour change potential is important to guide appropriate app choice for consumers and health professionals. There is an urgent need for a quick and usable tool to evaluate apps, which may increase LBP self-management app recommendation by health professionals and uptake by people with LBP.

This study has numerous methodological strengths, including national dissemination using a variety of paid and free online methods. The tools used in the survey were reliable and valid, allowing for a high level of confidence in the results. When compared with the national data from the 2022 National Health Survey (NHS) [5], the mean age of the study participants aligned with the age range of those experiencing the highest rates of LBP (55–74 years). Mean pain levels of study participants also aligned with NHS data and those in lower SES areas experienced increased burden of disease which aligned with the below standardised SEIFA mean for study participants. There were also some differences. This study had a high rate of female participants (74%); however, most health expenditure for back problems was for females (13% greater than for males), particularly those over 45 years of age. A lower percentage of this sample (45%) were employed compared to the NHS (71%).

There were also some limitations. Although the number of app users was small, it aligns with current trends [26]. The small sample size of app users limited inferential analysis and meant that it was not possible to explore associations between participant characteristics and app use variables due to low power. However, we were able to descriptively explore participant preferences and perceptions of smartphone apps for the self-management of LBP. These results should be interpreted with caution. The use of nonprobability sampling such as convenience/social and purposive sampling could have resulted in self-report bias, with these methods leading to over-representation of those who are digitally literate and health conscious and under-representation of those with limited access or interest in digital health content. This may limit the generalisability of results for the broader population.

## 5. Conclusion

The development of high-quality smartphone apps that can assist with self-management and behaviour change should be a focus of future app development. For currently available consumer-level apps, the assessment of app quality and self-management and behaviour change potential is an important future direction for research, guiding optimal app selection for people with LBP and health professionals. Adoption of informed and appropriate app use by health professionals in primary healthcare offers a novel LBP management option with potential to improve current LBP management and outcomes.

## Figures and Tables

**Table 1:** Participant characteristics for whole sample and app users.

	Whole sample (*n* = 136)	App users (*n* = 17)
Gender *n* (%) (female)	100 (73.5%)	13 (76.5%)
Age (years) mean ± SD (range)	55.5 ± 14.5 (18–80)	55.8 ± 15.7 (19–80)
English 1^st^ language *n* (%)	129 (94.9%)	15 (88.2%)
Aboriginal or Torres Strait Islander *n* (%)	5 (3.7%)	3 (17.7%)
Country born *n* (%)		
Oceania (Australia, New Zealand)	98 (72.1%)	13 (76.5%)
Europe	23 (16.9%)	1 (5.9%)
Asia	7 (5.1%)	1 (5.9%)
North America	2 (1.5%)	1 (5.9%)
Africa	6 (4.4%)	1 (5.9%)
Country mother born *n* (%)		
Oceania (Australia, New Zealand)	85 (62.5%)	12 (70.6%)
Europe	38 (27.9%)	4 (23.5%)
Asia	8 (5.9%)	1 (5.9%)
North America	1 (0.7%)	—
Africa	4 (2.9%)	—
Country father born *n* (%)		
Oceania (Australia, New Zealand)	82 (60.3%)	12 (70.6%)
Europe	41 (30.1%)	3 (17.7%)
Asia	7 (5.1%)	1 (5.9%)
North America	2 (1.5%)	1 (5.9%)
Africa	4 (2.9%)	—
Highest education *n* (%)		
High school not completed	16 (11.8%)	2 (11.8%)
High school completed	15 (11.0%)	1 (5.9%)
TAFE or trade school	29 (21.3%)	3 (17.7%)
Bachelor degree	27 (19.9%)	3 (17.7%)
Graduate certificate or diploma	29 (21.3%)	3 (17.7%)
Master's or doctoral degree	20 (14.7%)	5 (29.4%)
Employment status *n* (%)		
Employed	61 (44.9%)	6 (35.3%)
Unemployed	75 (55.1%)	11 (64.7%)
Retired	44 (32.4%)	7 (41.2%)
Looking for work	7 (5.2%)	1 (5.9%)
Student	6 (4.4%)	1 (5.9%)
Not looking for work	5 (3.7%)	—
Disability support pension	5 (3.7%)	2 (11.8%)
Carer	3 (2.2%)	—
Home duties	2 (1.5%)	—
Other	3 (2.2%)	—
SEIFA		
Disadvantage score mean ± SD (range)	995.6 ± 46.7 (906 – 1115)	1015 ± 41.9 (954 – 1092)
Decile score mean ± SD (range)	6.6 ± 2.4 (2–10)	7.6 ± 2.0 (4–10)
Percentile score mean ± SD (range)	61.6 ± 24.7 (14–99)	72.7 ± 19.8 (37 – 98)
Personality trait scores mean ± SD (range)		
Extroversion (^∗^norm = 4.4)	3.7 ± 1.5 (1–7)	3.5 ± 1.6 (1–7)
Agreeableness (^∗^norm = 5.2)	5.3 ± 1.1 (2.5–7)	4.8 ± 1.1 (2.5–7)
Conscientiousness (^∗^norm = 5.4)	5.1 ± 1.4 (1–7)	5.4 ± 0.9 (3.5–7)
Emotional stability (^∗^norm = 4.8)	4.3 ± 1.5 (1–7)	4.8 ± 1.5 (2–7)
Openness (^∗^norm = 5.4)	4.8 ± 1.1 (2–7)	4.9 ± 1.0 (3.5–7)
Current LBP *n* (%)	127 (93.4%)	17 (100%)
Average LBP severity mean ± SD (range)	6.3 ± 1.8 (3–10)	6.1 ± 1.9 (3–10)
Duration of LBP *n* (%)		
< 6 weeks	33 (24.3%)	3 (17.7%)
6–12 weeks	12 (8.8%)	—
> 12 weeks	91 (66.9%)	14 (82.4%)
Recurrence of LBP *n* (%)		
Constantly-daily	69 (58.0%)	12 (70.6%)
< Daily-monthly	20 (16.8%)	2 (11.8%)
<Monthly-yearly/randomly/activity dependent	30 (25.2%)	3 (17.7%)

^∗^Normal population [45].

**Table 2:** Frequency of use of self-management options for LBP.

	Never/rarely *n* (%)	Occasionally *n* (%)	Frequently/very frequently *n* (%)
Advice from health professionals (*n* = 126)	27 (21.5)	51 (40.5)	**48 (38.1)**
Internet information (*n* = 125)	62 (49.6)	47 (37.6)	16 (12.8)
Exercise (*n* = 126)	27 (21.5)	40 (31.7)	**59 (46.8)**
Manual therapies (*n* = 126)	37 (29.4)	52 (41.3)	37 (29.4)
Psychological therapies with physical programs (*n* = 126)	93 (69.0)	26 (20.6)	13 (10.3)
Return to work programs (*n* = 126)	114 (90.4)	9 (7.1)	3 (2.4)
Pharmacological management (*n* = 132)	48 (38.1)	31 (24.6)	**47 (37.3)**
Other (*n* = 98)	76 (77.5)	6 (6.1)	16 (16.3)

*Note:* Bold = most frequently used.

**Table 3:** Associations between demographic factors and self-efficacy and self-management.

	Self-efficacy (NGSE) (*n* = 136) Standardised Coef. [95%CI] (*p* value) (adjusted *R*^2^)	Self-management (SMST) (*n* = 127) Standardised Coef. [95%CI] (*p* value) (adjusted *R*^2^)
Gender	(*n* = 135) 0.118 [−0.069, 0.521] (*p* = 0.171) (0.007)	(*n* = 126) 0.253 [0.745, 3.641] **(p**=0.004**) (0.57)**
Age (years)	(*n* = 135) 0.066 [-0.135, 0.305] (*p*=0.445) (−0.003)	(*n* = 126) 0.252 [0.505, 2.674] **(p**=0.004**) (0.056)**
English 1st language	0.134 [0.110, 0.832] (*p*=0.119) (0.011)	0.081 [−0.297, 3.123] (*p* = 0.367) (−0.01)
Aboriginal or Torres Strait Islander	(*n* = 134) 0.075 [−0.558, 1.273] (*p* = 0.387) (−0.002)	(*n* = 125) 0.086 [−0.518,4.269] (*p* = 0.340) (−0.001)
Birth country	0.111 [−0.111, 0.684] (*p* = 0.198) (0.005)	(*n* = 126) −0.014 [−2.293, 1.797] (*p* = 0.876) (−0.008)
Maternal birth country	0.148 [0.026, 0.784] (*p* = 0.085) (0.015)	0.044 [−1.538, 2.363] (*p* = 0.622) (−0.006)
Paternal birth country	0.142 [−0.082, 0.821] (*p* = 0.100) (0.013)	0.023 [−2.499, 3.234] (*p* = 0.800) (−0.007)
Highest education	−0.095 [−0.421, 0.100] (*p* = 0.272) (0.002)	(*n* = 126) −0.120 [−2.324, 0.470] (*p* = 0.180) (0.006)
Employment status	−0.151 [−0.500, 0.044] (*p* = 0.079) (0.016)	0.091 [−0.744, 2.064] (*p* = 0.309) (0.000)
SES SEIFA percentile (1%–100%)	−0.221 [−0.012, −0.002] **(*p* = 0.010) (0.042)**	0.026 [−0.023, 0.034] (*p* = 0.783) (−0.007)
Personality traits		
Extroversion score (*n* = 126)	0.208 [0.036, 0.368]^b^**(*p* = 0.015) (0.076)**	0.220 [0.129, 1.031] **(*p* = 0.010) (0.041)**
Agreeableness (*n* = 127)	0.269 [0.101, 0.423]^b^**(*p* = 0.002) (0.045)**	0.051 [-0.478,0.869] (*p* = 0.573) (−0.005)
Conscientiousness (*n* = 127)	0.444 [0.293, 0.573]^b^**(*p* < 0.001) (0.268)**	0.404 [0.709, 1.574] **(*p* < 0.001) (0.157)**
Emotional stability (*n* = 127)	0.383 [0.225, 0.522]^b^ (***p* < 0.001) (0.197)**	0.462 [0.779, 1.606] **(*p* < 0.001) (0.207)**
Openness (*n* = 127)	0.380 [0.221, 0.519]^b^**(*p* < 0.001) (0.120)**	0.314 [0.535, 1.652] **(*p* = 0.002) (0.091)**
LBP characteristics		
Current LBP (*n* = 127)	0.039 [−0.271, 0.488] (*p* = 0.651) (−0.006)	−0.098 [−6.167, 2.871] (*p* = 0.396) (0.002)
Average LBP severity (*n* = 127)	−0.068 [−0.107, 0.046] (*p* = 0.432) (−0.003)	−0.215 [−0.892, −0.055] **(*p* = 0.029) (0.039)**
Duration of LBP (*n* = 127	−0.213 [−0.341, −0.027] **(*p* = 0.013) (0.038)**	−0.032 [−0.990, 0.706] (*p* = 0.717) (−0.007)
Recurrence of LBP (*n* = 112)	−0.266 [−0.394, −0.118] **(*p* = 0.003) (0.063)**	0.048 [−0.731, 1.075] (*p* = 0.614) (−0.007)

*Note:* BOLD = significant *p* = 0.05. Age categories (years)–18–34, 35–64, 65+.

Abbreviations: NGSE  =  new general self-efficacy score, SMST = self-management self-test score.

## Data Availability

The data that support the findings of this study are available from the corresponding author upon reasonable request.
